# Effects of addition of 2-fucosyllactose to infant formula on growth and specific pathways of utilization by *Bifidobacterium* in healthy term infants

**DOI:** 10.3389/fnut.2022.961526

**Published:** 2022-09-23

**Authors:** John C. Wallingford, Pernille Neve Myers, Cynthia M. Barber

**Affiliations:** ^1^Nutrispectives, LLC, Spokane, WA, United States; ^2^Clinical Microbiomics, Copenhagen, Denmark; ^3^Perrigo Nutritionals, Charlottesville, VA, United States

**Keywords:** bifidobacteria, infant formula, fucosyllactose, growth, gene expression

## Abstract

Oligosaccharides in human milk support health *via* intestinal microbiome. We studied effects of addition of 2-fucosyllactose (2′FL) to the infant formula on infant growth, occurrence of adverse events (AE), and infant microbiome, including expression of microbial genes that metabolize 2′FL. Our hypothesis was that while 2′FL would not affect growth, it would cause changes in microbiome metabolism. In a double-blinded randomized controlled study fashion, the infant formula ± 2′FL or human milk was fed to healthy term infants for 16 weeks. Fecal samples obtained at baseline and week 16 were analyzed for microbial populations, metagenomic species concept (MGS), and genetics of gut metabolic modules (GMMs). There were no effects of addition of 2′FL on growth or AEs. There were no significant differences by feeding group in MGS richness or Shannon diversity at baseline, but formula groups each had significantly greater richness (*p* < 0.05) and diversity (*p* < 0.05) after 16 weeks of feeding than the breastfed group. While two glycosyl hydrolase (GH) families (GH42 and GH112) were significantly increased, two other GH families (GH20 and GH2) were significantly decreased in the test formula group compared to the control formula group; although modest, addition of 2′FL resulted in changes in microbiome in the direction of breastfed infants, consistent with internal metabolism of HMOs by *Bifidobacterium.*

## Introduction

Human milk oligosaccharides (HMOs) are the third most abundant constituent of human milk after lactose and fat, and more of protein, contributing some 8–15% to the total (1). The proportion of fucosylated, sialylated, and non-fucosylated neutral HMOs in term human milk has been reported as 35–50, 12–14, and 42–55%, respectively (2). The carbohydrate composition of human milk has been difficult to mimic in formulas because after lactose, human milk carbohydrates are predominantly oligosaccharides, for which until recently, there was no readily available ingredient source.

There is a presumption that a high concentration of 2-fucosyllactose (2′FL) in human milk has resulted from evolutionary pressures and conveys benefit to infants, distinct from other mammalian species because sugar is not detected in the milk of other species. One randomized controlled trial reported fewer infection-related adverse events (AEs) among infants fed formula containing 2′FL than among infants fed the standard formula (3). A second single arm study showed resolution of most symptoms in most infants fed an extensively hydrolyzed formula because of their medical history and clinical symptoms (4). HMOs including 2′FL may act as prebiotics, selectively metabolized by bacteria considered beneficial to humans (5, 6). HMOs inhibit bacterial adhesion to intestinal epithelial cell surface epitopes, offering an alternative binding site (7, 8). 2′FL inhibits attachment of pathogens like *Campylobacter jejuni*, enteropathogenic *Escherichia coli*, *Salmonella enterica*, *Pseudomonas aeruginosa*, RSV, and influenza virus to human intestinal and respiratory cell lines (9, 10). On the other hand, secretors have been reported to be more likely infected by rotavirus and norovirus (11). 2′FL inhibits binding of a wide array of glycans to DC-SIGN, a domain of dendritic cells and macrophages involved in bacterial and viral recognition and internalization, suggesting milk 2′FL modulates gastrointestinal immune monitoring (12). Pooled HMOs or 2′FL alone was reported to suppress inflammation following enterotoxigenic *E. coli* (ETEC) or *E. coli* infection, suggesting HMOs are part of the innate immune system (13). Finally, HMOs may be absorbed to a small extent and have systemic actions. Isolated peripheral blood mononuclear cells from 10-day-old piglets produced more IL-10 when exposed to isolated pooled HMOs and proliferated less when exposed to 2′FL (14). Addition of 2′FL to the feed of infant rodents increased hippocampal long-term potentiation in mice and rats (15).

This clinical study examined effects of 2′FL on growth of healthy term infants, in accordance with U.S. FDA regulatory requirements, and the effects of addition of 2′FL on the infant microbiome. We report that while 2′FL had no discernible effect on growth or incidence of adverse effects, there was a selective enrichment of *Bifidobacterium*, in particular *Bifidobacterium* spp. that have genes for intracellular metabolism of 2′FL, supporting the hypothesis of prebiotic effects of 2′FL. In addition, because approximately 20% of the human population lacks the gene to synthesize 2′FL (15), we examined the response of the microbiome as a function of the genotype for maternal and infant fucosyltransferase, allowing an assessment of the relative importance of exogenous and endogenous provision of 2′FL.

## Materials and methods

This study was performed in compliance with applicable U.S. FDA regulations (21 CFR Parts 50, 54, 56, and 312), the ethical principles of the Declaration of Helsinki, all applicable International Council for Harmonization of Technical Requirements for Pharmaceuticals for Human Use (ICH) Good Clinical Practice (GCP) guidelines, and all local laws and regulations concerning clinical studies. The Institutional Review Board (IRB)-approved informed consent form (ICF) was reviewed and signed by each subject’s parent(s) or legal representative(s) prior to enrollment.

This was a double-blinded randomized controlled study with two formula-fed arms and a reference breastfed^1^ group. The infant formula or human milk was fed to healthy term infants for a period of 16 weeks upon entering the study as their sole source of nutrition. The formulas consisted of a commercial product (control) and the same formula also containing HMO 2′FL at 1 g/L (test). Altogether, a total of nine study sites in the United States (*n* = 8) and Honduras (*n* = 1) were selected.

Eligible infants were enrolled on or before 28 days of age and fed a study-assigned formula or were breastfed for 16 weeks. The weight, length, and head circumference of the infants were measured at baseline and 2, 4, 6, 8, 12, and 16 weeks of feeding. AEs were recorded at monthly in-person caregiver visit to the clinic and by telephone between in-person visits. Fecal samples were obtained at baseline when available and at week 16 for all subjects and analyzed for microbial populations. Information on AEs and new/change of medications was collected at in-person visits at weeks and by telephone interviews between in-person visits.

### Subjects and formulas

Healthy term (37–42 weeks of gestation) infants of either sex with a birth weight between the 5th and 95th percentiles and APGAR scores of 7 or greater at birth were eligible. The study formula assigned to each subject was determined by a computer-generated randomization. The investigator, study staff, study monitors, data analysis/management personnel, and parents were blinded to the subject formula assignments. The infants were fed *ad libitum*. The control formula was a commercial whey-dominant cow milk-based infant formula with canola oil in the fat blend and contained the following optional ingredients: galactooligosaccharide (GOS), fructooligosaccharide (FOS) docosahexaenoic acid (DHA), arachidonic acid (ARA), lutein, and nucleotides. The test formula was identical to the control formula, except that it included 2′FL (1 g/L, from Jennewein, now owned by CHR Hansen, that was synthesized by genetically modified *E. coli* BL21 (DE3), #1540, per GRAS Notification #571) at the expense of the same amount of GOS. Feeding compliance was determined from caregiver diary during in-person visits and *via* phone/electronic contact at scheduled intervals between in-person visits.

Anthropometric measurements of weight, length, and head circumference were obtained at days 1, 14, 28, 42, 56, 84, and 116 using an “All-In-One Infant Station” (Perspective Enterprises, Portage, MI). Weight was recorded to the nearest 5 g. The infants were weighed naked without clothes or diaper. To the greatest extent possible, the same personnel who obtained the measurements at visit 1 was to obtain them at visits 3, 5, 7, 9, and 11. Length was obtained to 0.1 cm (1 mm). Length gain was measured as total centimeters gained over the 16-week feeding period (length at week 16 minus length at day 1). The head circumference was measured to 0.1 cm (1 mm) using a circumference tape.

The primary efficacy variable was weight gain, measured as total grams gained over the 16-week feeding period (weight at week 16 minus weight at day 1), analyzed by mean daily weight gain (calculated as total weight gain divided by the total number of days). The primary comparison was between the test and control formula groups. Weight gain in the test formula group was also compared to weight gain in the reference breastfed infants.

### Sample size statistics

Based on a previous similar study (3), a non-inferiority margin of the daily gain weight of –3 g/day (test vs. control) and *SD* = 4.5 g/day (16) were used for the sample size calculations. For a significance level of 2.5% (one-sided test), a sample size of 37 subjects per treatment arm or a total of 74 subjects in the PP population provided 80% power to show non-inferiority between test and control formula groups for mean daily weight gain. For a 90% statistical power, a sample size of 49 subjects per feeding formula arm or 98 subjects completing the study in the PP population were required. Considering a dropout rate of about 25% from the ITT to PP population, approximately 66 subjects were needed to be enrolled in each formula feeding group.

Analysis of covariance (ANCOVA) was used to analyze the primary endpoint of mean daily weight gain (g/day) between the test and control formula groups. The model included formula group, gender, and interaction formula-by-gender as fixed effects, and baseline weight as a covariate. Subjects from all three dietary groups were included in the model.

### Adverse events

An AE was defined as any untoward medical occurrence associated with the use of the investigational product whether or not the event was considered product related, and was coded using MedDRA version 20.0. An AE could also include the exacerbation of pre-existing conditions and intercurrent illnesses. Potential AEs were collected by interview at study visits and by telephone calls. Serious adverse events (SAEs) not resolved within 30 days of the final study visit were followed until they resolved or were considered to be chronic.

### Fecal samples

Stool samples were collected by caregivers in provided collection tubes (DNA Genotek, Ottawa, Canada), were brought to clinics, and were stored by study personnel. After obtaining informed consent on day 1, if a stool sample was available, 1 g of stool was collected and was subsequently frozen-stored (–20°C). Day 112 samples were brought to study site fresh, were coded, and frozen until shipment to Clinical Microbiomics (Copenhagen, Denmark) for analysis.

### Microbiome

DNA was extracted from ∼0.1 g aliquots of the fecal samples using the NucleoSpin 96 Soil (Macherey-Nagel) kit. Fragmented DNA was used for library construction using a NEBNext Ultra Library Prep Kit for Illumina (New England Biolabs). Quantitative real-time PCR (qPCR) was used to determine the concentration of the final library before sequencing. The library was sequenced using 2 × 150-bp paired-end sequencing on the Illumina platform. The metagenomic analysis was performed using the metagenomic species (MGS) concept (17) and the Clinical Microbiomics human gut MGS database. For taxonomic abundance profiling, we used the Clinical Microbiomics HGMGS version HG4.D.1 set of 2095 metagenomic species (MGS), each represented by a set of genes with highly coherent abundance profiles and base compositions in the 12,170 metagenomes, which includes 481 from infants, 9,428 publicly available metagenomes (18), and 3,567 publicly available genome assemblies from isolated microbial strains. Individual high-quality non-host reads were mapped to a gene if the mapping quality (MAPQ) was ≥ 20, and the reads aligned with ≥ 95% identity over ≥ 100 base pairs.

### Genes for 2-fucosyllactose metabolism

GMMs, conserved metabolic pathways, were assessed using KEGG Orthology (KO) identifiers (19). Genes in the fucose pathway: K02431 (FumB), K07046 (FumD), K18334 (FumE), K22397 (FumF), and K02429 (FumP, fucose transporter) (20) were mapped to KO identifiers that were also found in the gene catalog. An MGS was considered to have the functional potential to grow on fucose using the *Bifidobacterium* pathway if at least three of the five genes were present in the MGS. Glycosyl hydrolases GH2, GH20, GH29, GH33, GH42, GH95, GH112, and GH136 (21) were classified using dbCAN2 (v. 2.0.6) (22) based on homology search using HMMER (v. 3.2.1). Analyses of GH families were conducted for total gene abundance and separately for genes with a signal peptide for transmembrane transport of the gene product (external strategy) or not (internal strategy). Signal peptides were annotated using the signalP webserver (v. 5.0) (23).

### Microbiome statistics

Statistical analysis of differences in microbiome composition between feeding groups was performed in R (v. 4.1.1). The Mann–Whitney U test was used for comparisons. The generalized fold change in gene-level abundances was calculated as previously described (24).

## Results

A total of 221 subjects were enrolled; 66 subjects received the test formula, 66 subjects received the control formula, and 89 subjects were breastfed. Most demographic and baseline characteristics were comparable among the three dietary groups (Table 1). The mean (*SD*) age of subjects at enrollment was 14 days; the proportion of male and female infants among groups was comparable, with a slight excess of female infants in each group. Most infants were white, predominantly Hispanic or Latino, followed by Black and African American. The mode of delivery of most of the infants was vaginal, with a similar proportion between formula groups but slightly less than among infants who were breastfed. Weight, length, and head circumference were similar at enrollment across diet groups as well.

**TABLE 1:** Demographic and baseline characteristics (safety population).

	Test formula	Control formula	Breastfed	Total
	
	(*n* = 66)	(*n* = 66)	(*n* = 89)	(*n* = 221)
Age (d), mean (*SD*)	14.1 (6.72)	14.3 (7.05)	13.6 (6.35)	14.0 (6.66)
Male (*n*)	32 (48%)	28 (42%)	42 (47%)	102 (46%)
White (*n*)	53 (80%)	49 (74%)	75 (84%)	177 (80%)
Hispanic or Latino (*n*)	42 (64%)	39 (59%)	27 (30%)	108 (49%)
Black or African American (*n*)	7 (11%)	9 (14%)	9 (10%)	25 (11%)
Other/mixed	6 (9%)	8 (12)	5 (6%)	19 (9%)
Vaginal delivery	44 (67%)	45 (68%)	67 (75%)	156 (71%)
Weight mean, g (*SD*)	3,588 (511)	3,587 (453)	3,590 (421)	3,589 (457)
Length mean, cm (*SD*)	51.9 (3.0)	51.8 (2.0)	52.0 (2.2)	51.9 (2.4)
Head circumference mean, cm (*SD*)	35.8 (1.5)	35.7 (1.3)	35.6 (1.4)	35.7 (1.4)

A total of 176 (79.6%) subjects completed the study (Table 2). The number of subjects who discontinued the study was higher in the control formula group (37.9%) than in the breastfed group (11.2%) and the test formula group (15.2%).

**TABLE 2:** Subject disposition.

	Test formula	Control formula	Breastfed	Total
	
	*n* (%)	*n* (%)	*n* (%)	*n* (%)
Enrolled/safety population	66 (100.0)	66 (100.0)	89 (100.0)	221 (100.0)
Per protocol population	47 (71.2)	35 (53.0)	78 (87.6)	160 (72.4)
Subjects who completed the study	56 (84.8)	41 (62.1)	79 (88.8)	176 (79.6)
Subjects who discontinued from the study	10 (15.2)	25 (37.9)	10 (11.2)	45 (20.4)
Reasons for discontinuation from study				
Non-compliance	0	0	2	2
Lost to follow-up	2	7	1	10
Adverse event	2	4	0	6
Parent(s)/legal guardian request	6	11	5	22
Other	0	3	2	4

Overall, the primary reasons of study discontinuation were requests by parents/legal guardian (*n* = 22, 48.9%) and subjects being lost to follow-up (*n* = 10, 22.2% subjects); 13.3% subjects experienced AEs that led to study discontinuation; these AEs included vomiting, abdominal pain, gastroesophageal reflux disease (GERD), constipation, and spitting up. The most frequently reported protocol violations were early termination of subjects (last subject visit was < 4 months from visit 1) (20.4% subjects) and COVID-19 (6.8% subjects).

In total, three subjects fed the test formula experienced AEs that led to discontinuation of the study formula: a moderate AE of projectile vomiting was considered possibly related to the study formula; a moderate AE of infant irritability and a severe AE of infantile spitting up were considered possibly related to the study formula, and one moderate AE of abdominal pain was considered probably related to the study formula.

A total of seven subjects fed the control formula experienced AEs, which led to discontinuation of the study formula; two AEs were considered likely or definitely related to formula, whereas the others were considered unlikely related. The seven subjects had a moderate AE of GERD; a moderate AE of constipation; moderate AEs of flatulence and GERD and a severe AE of crying; a mild AE of diarrhea; mild AEs of abdominal pain and constipation; a mild AE of infantile spitting up; and a mild AE of abdominal pain.

Despite greater attrition from the control group, the per protocol subjects in the two formula groups were similar in demographics and anthropometrics at enrollment (Table 3).

**TABLE 3:** Demographic and baseline characteristics (PP population).

Characteristic	Test formula	Control formula	Breastfed	Total
	
	*n* = 47	*n* = 35	*n* = 78	*n* = 160
Age in days, mean (*SD*)	13.7 (6.9)	14.1 (7.6)	13.7 (6.6)	13.8 (6.9)
Male (%)	22 (46.8)	14 (40.0)	36 (46.2)	72 (45.0)
Vaginal delivery (%)	33 (70.2)	25 (71.4)	60 (76.9)	118 (73.8)
Weight, g, mean (*SD*)	3,561 (539)	3,640 (485)	3,595 (429)	3,595 (474)
Length, cm, mean (*SD*)	51.7 (3.2)	52.0 (1.8)	52.1 (2.1)	52.0 (2.4)
Head circumference, cm, mean (*SD*)	35.6 (1.4)	35.7 (1.3)	35.5 (1.4)	35.6 (1.4)

### Growth

Growth of the per protocol infants fed the test formula, measured as average daily weight gain, was not inferior to the growth of infants fed the control formula (Table 4). The mean daily weight gain was 30.6 g for the test formula group, 30.3 g for the control formula group, and 29.0 g for the breastfed group. The lower bound of the 95% CI of difference between the least squares (LS) means of test and control formula groups was –2.8, greater than non-inferiority margin of –3 g/day. The lower bound of the 95% CI of difference between the LS means of test formula and breastfed groups was –0.6. The weight gain among the formula-fed subjects in Honduras was slightly more than that among U.S. infants, but that difference was smaller than the non-inferiority margin of 3.0 g/d and smaller than the difference between the breastfed Honduran subjects and breastfed U.S. subjects.

**TABLE 4:** Analysis of mean daily weight gain (grams) (PP population).

Statistic	Test formula (*n* = 47)	Control formula (*n* = 35)	Breastfed (*n* = 78)
Mean (*SD*)	30.5 (6.1)	29.9 (7.3)	28.4 (8.1)
Median (Min, Max)	30.0 (13.5, 49.3)	28.0 (18.9, 53.9)	27.1 (12.2, 46.5)
LS mean (SE)	30.6 (1.0)	30.3 (1.2)	28.6 (0.9)
95% CI of LS mean	28.6, 32.6	27.9, 32.7	27.0, 30.2
LS mean difference test vs. Control or test vs. Breastfed		0.3	2.0
95% CI of LS mean difference		–2.8, 3.4	–0.6, 4.6

Only three subjects in the test group had any non-study formula recorded, each instance a feeding of four ounces of a formula; two subjects in the control group were recorded as having a non-study formula, one a serving of four ounces, and the three servings of six total servings on the last study day record.

Similar results were obtained from supportive analysis in the ITT population. Mean daily weight gain was 29.5 g for the test formula, 28.8 g for the control formula, and 28.4 g for the breastfed group. The lower bound of the 95% CI of difference between the LS means of the test formula and control formula groups (–2.3), and that between the test formula and breastfed groups (–1.4) were both greater than non-inferiority margin of –3 g/day.

Mean (LS mean) length gain (change from baseline) at visit 11 was 12.2 cm for the test formula group, 11.6 cm for the control formula group, and 11.3 cm for the breastfed group. Mean (LS mean) gain in the head circumference (change from baseline) at visit 11 was 6.2 cm for the test formula group, 6.2 cm for the control formula group, and 6.1 cm for the breastfed group. There were no significant differences in length gain or head circumference gain between the test and control formula groups and between the test formula and breastfed groups at any time. The 95% CIs of difference between the LS means of the test formula and control formula and between LS means of the test formula and breastfed groups for comparisons at each visit included 0, indicating that there was no statistically significant difference in gain in length or head circumference between the test and control formula groups, or between the test formula and breastfed groups at any measurement time. The analysis of the ITT population also found no differences between formula groups or between the test and BF groups in gains at any time.

Because of difficulty in enrollment, the protocol was revised to allow subjects of age up to 29 days of life to be enrolled. The same proportion of formula-fed infants in the test group were enrolled after the protocol change (57%) as before the change (58%). The weight gain of infants enrolled before day 15 and that of infants enrolled on or after day 15 showed that there was no difference in gain as a function of age at enrollment (Table 5). The mean weight gain for the two formula groups was about less than 3 g/d in each subgroup, and the LS mean difference in each subgroup was within the 95th percentile confidence interval for the three diets. Similarly, comparisons of LS mean differences between the test and BF groups found all differences to be within the 95th centile confidence interval (data not shown). We did not analyze the differences within a feeding group over time, although inspection shows very little difference in either formula groups, where among breastfed infants, there appeared to be lower weight gain of infants enrolled at later ages. We previously reported that among growth studies that had a reference BF group, formula-fed infants were commonly reported to have gained more weight in the 2nd and 3rd months than BF infants (25).

**TABLE 5:** Weight gain as a function of age at enrollment.

Age at enrollment	Test (g/d)	Control (g/d)	BF (g/d)
0–14 d	30.0^a^ ± 4.8 (23)	30.6 ± 8.8 (17)	31.0 ± 8.3 (37)
LS difference from control	–1.73^b^ [–6.6, 3.1]^c^		
15–21 d	30.9 ± 8.2 (18)	29.4 ± 5.2 (13)	26.8 ± 7.0 (37)
LS difference from control	1.56 [–3.3, 6.4]		
22–29 d	31.1 ± 3.1 (6)	29.1 ± 6.8 (5)	23.7 ± 9.1 (6)
LS difference from control	1.1 [–9.8, 12.0]		

^a^Mean ± SD; n in parentheses; ^b^least square mean difference from control, ^c^95% confidence interval in brackets.

### Safety data

The number of infants affected at any time during the study, by organ system, is shown in Table 6. This population is all infants enrolled and may underestimate the number of AEs for the control formula group because fewer subjects in the control group completed the study (62%) than the test group (85%) or breastfed group (89%). The majority of subjects experienced AEs that were either mild (23.5%) or moderate (17.6%). The majority of AEs in the three dietary groups were gastrointestinal disorders (22.6%), infections and infestations (19.9%), and skin and subcutaneous tissue disorders (13.1%). There was a slightly greater incidence of gastrointestinal disorders in the two formula groups than in the reference breastfed group, but the two formula groups had the same incidence (24%).

**TABLE 6:** Adverse events by system organ class and preferred term (safety population).

System organ class	Test formula	Control formula	Breastfed	Total
	
	(*n* = 66)	(*n* = 66)	(*n* = 89)	(*n* = 221)
	
	*n* (%)	*n* (%)	*n* (%)	*n* (%)
Number of subjects with at least one event	28 (42.4)	21 (31.8)	48 (53.9)	97 (43.9)
**Gastrointestinal disorders**	**16 (24.2)**	**16 (24.2)**	**18 (20.2)**	**50 (22.6)**
Abdominal pain	1 (1.5)	2 (3.0)	1 (1.1)	4 (1.8)
Constipation	4 (6.1)	5 (7.6)	1 (1.1)	10 (4.5)
Diarrhea	1 (1.5)	3 (4.5)	0	4 (1.8)
Flatulence	5 (7.6)	1 (1.5)	4 (4.5)	10 (4.5)
Gastrooesophageal reflux disease	3 (4.5)	3 (4.5)	5 (5.6)	11 (5.0)
Vomiting	2 (3.0)	1 (1.5)	2 (2.2)	5 (2.3)
**Infections and infestations**	**15 (22.7)**	**6 (9.1)**	**23 (25.8)**	**44 (19.9)**
Bronchiolitis	2 (3.0)	0	0	2 (0.9)
Candida infection	2 (3.0)	0	3 (3.4)	5 (2.3)
Conjunctivitis	0	0	4 (4.5)	4 (1.8)
Nasopharyngitis	5 (7.6)	1 (1.5)	2 (2.2)	8 (3.6)
Oral candidiasis	1 (1.5)	0	2 (2.2)	3 (1.4)
Otitis media	1 (1.5)	0	5 (5.6)	6 (2.7)
Upper respiratory tract infection	2 (3.0)	3 (4.5)	7 (7.9)	12 (5.4)
Viral infection	4 (6.1)	0	0	4 (1.8)
Viral upper respiratory tract infection	0	0	2 (2.2)	2 (0.9)
**Skin and subcutaneous tissue disorders**	**7 (10.6)**	**7 (10.6)**	**15 (16.9)**	**29 (13.1)**
**Respiratory, thoracic and mediastinal disorders**	**5 (7.6)**	**3 (4.5)**	**12 (13.5)**	**20 (9.0)**

Bold face indicates a significant difference in incidence among feeding groups, p < 0.05.

The overall incidence of reported AEs in formula-fed infants was higher in the U.S. (55%) than in Honduras (22%) subjects; the rates for formula-related events was 8% in the U.S. and 3% in Honduras subjects. There was no difference between formula groups in incidence rates of reported AEs in either country subjects. About two-thirds or the AEs reported were related to infection, which slightly higher in the U.S. than in Honduras subjects.

Overall, six subjects who completed the study experienced eight SAEs in the study, none were life-threatening or fatal, and none were considered related to the study formula. In the test formula group, one subject experienced two SAEs related to viral infection. In the breastfed group, one subject experienced a moderate SAE of parainfluenza virus infection; one subject experienced severe AEs of urinary tract infection and post-procedural hemorrhage; one subject experienced a severe SAE of pneumonia respiratory syncytial viral; one subject experienced a mild SAE of Sandifer’s syndrome; and one subject experienced a moderate SAE of poor feeding.

### Microbiome

A total of 162 fecal samples from 133 infants were successfully sequenced using shotgun metagenomic sequencing, of which 55 samples were collected at the initial visit and 107 were collected at the final visit. The samples were profiled against a catalog of metagenomic species (MGS) identified using canopy clustering (17).

**FIGURE 1:**
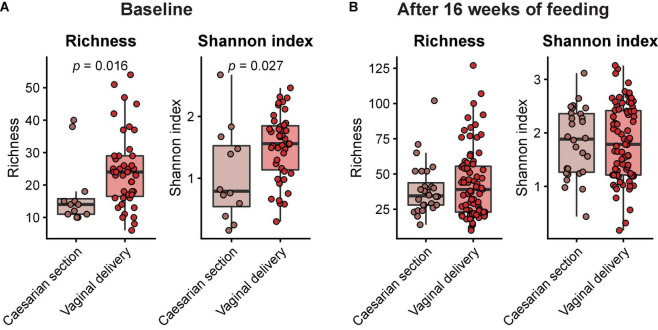
Box plots of MGS richness and Shannon diversity index at baseline **(A)** and after 16 weeks of feeding **(B)** in infants born by either caesarian section or vaginal delivery. Horizontal lines indicate median; box boundaries indicate the interquartile range; whiskers represent values within 1.5 × the interquartile range of the first and third quartiles. Statistics, Mann–Whitney *U*-test. *P*-values < 0.05 are shown.

**FIGURE 2:**
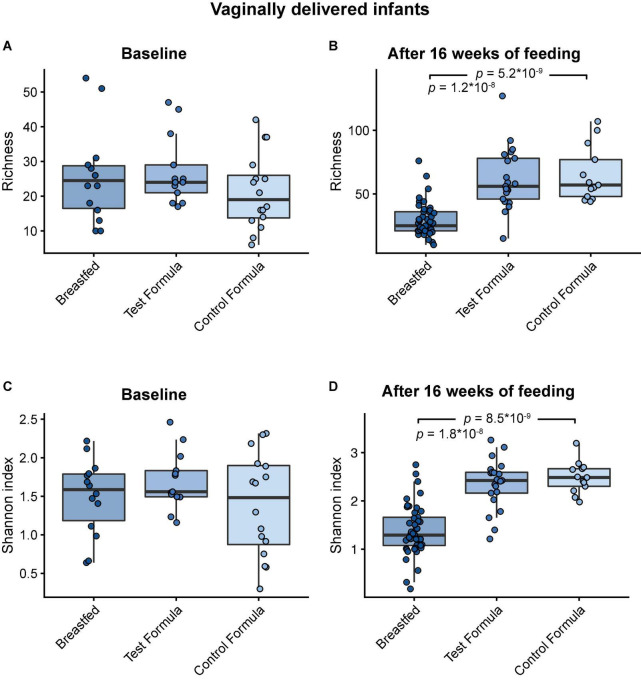
Box plots of MGS richness and Shannon diversity index at baseline in vaginal delivered infants **(A,B)** and after 16 weeks of feeding in infants **(C,D)** in the breastfed, test formula, or control formula feeding group. Horizontal lines indicate the median; box boundaries indicate the interquartile range; whiskers represent values within 1.5 × the interquartile range of the first and third quartiles. Statistics: Mann–Whitney *U*-test. *P*-values < 0.05 are shown.

**FIGURE 3:**
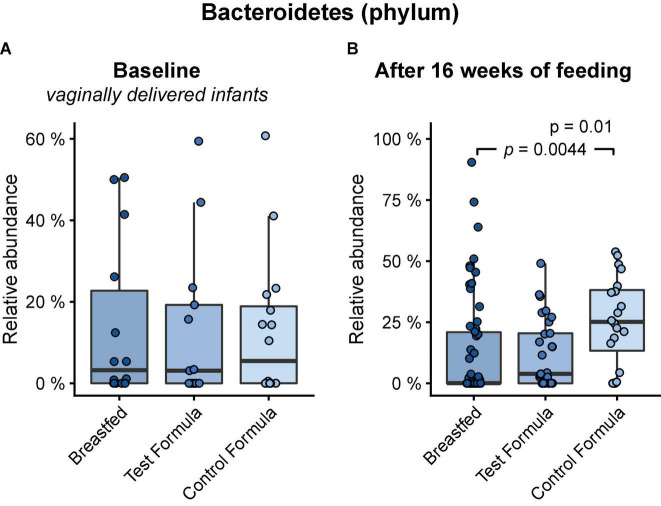
Box plots of the relative abundance of Bacteroidetes at baseline in vaginally delivered infants **(A)** and after 16 weeks of feeding in infants **(B)** in the breastfed, test formula, or control formula-feeding group. Horizontal lines indicate the median; box boundaries indicate the interquartile range; whiskers represent values within 1.5 × the interquartile range of the first and third quartiles. Statistics: Mann–Whitney *U*-test. *P*-values are shown when *Q* (FDR) < 0.1.

Across feeding treatments, vaginally delivered infants had greater MGS richness (*p* = 0.016) and Shannon diversity (*p* = 0.027) in the initial samples than infants delivered by cesarean section, although there was no difference in these measures in the week 16 samples (Figure 1). There were no significant differences by feeding group in MGS richness or Shannon diversity at baseline in the subset of infants, whether born vaginally or among all infants (Figure 2). The formula groups each had significantly greater richness (*p* < 0.05) and Shannon diversity (*p* < 0.05) after 16 weeks of feeding than the breastfed group but did not differ from each other. Likewise, we found no significant differences by feeding group at any of the taxonomic levels at baseline in the subset of vaginally delivered infants after adjusting for multiple testing (*Q* < 0.1, Supplementary Table 1), while numerous taxa were significantly differentially abundant between the breastfed and either of the formula groups after 16 weeks of feeding (*Q* < 0.1, Supplementary Table 2). Bacteroidetes (phylum) was the only taxa that was significantly differentially abundant between the test formula and control formula groups (*Q* < 0.1), where it was more abundant in the control formula group after 16 weeks of feeding (Figure 3). Bacteroidetes was also more abundant in the control formula group than in the Breastfed group, whereas there was no difference between the breastfed and test formula groups.

**FIGURE 4:**
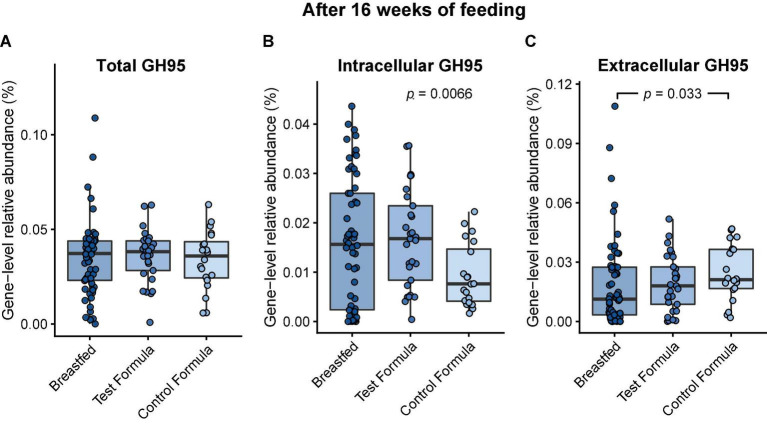
Box plots of the relative gene-level abundance of GH95 after 16 weeks of feeding in infants in the three feeding groups: breastfed, test formula, and control formula. Comparisons between the feeding groups were carried out for total GH95 **(A)**, GH95 encoding a signal peptide corresponding to the external strategy **(C)**, and GH95 which did not encode a signal peptide **(B)**. Horizontal lines indicate the median; box boundaries indicate the interquartile range; whiskers represent values within 1.5 × the interquartile range of the first and third quartiles. Statistics: Mann–Whitney *U*-test. *P*-values < 0.05 are shown.

Utilization of the HMO 2′FL by the gut microbiome requires cleaving the monosaccharide fucose from the lactose core. In the infant microbiome, this is achieved by species that express 1,2-α-fucosidase belonging to the glycosyl hydrolase family 95 (GH95) (21). There were no significant differences in the relative gene-level abundances of GH95 between any of the feeding groups after 16 weeks of feeding (Figure 4A). However, GH95 may be expressed with or without a signal peptide corresponding to, respectively, the external or internal strategy for HMO utilization (26). The addition of 2′FL to the test formula was associated with a significant increase (*p* = 0.0066) in the gene-level abundance of GH95 expressed without a signal peptide after 16 weeks of feeding (Figure 4B, “internal”). The control formula was associated with a significant increase (*p* = 0.033) in the abundance of GH95 expressed with a signal peptide compared to the breastfed group (Figure 4C, “external”).

**FIGURE 5:**
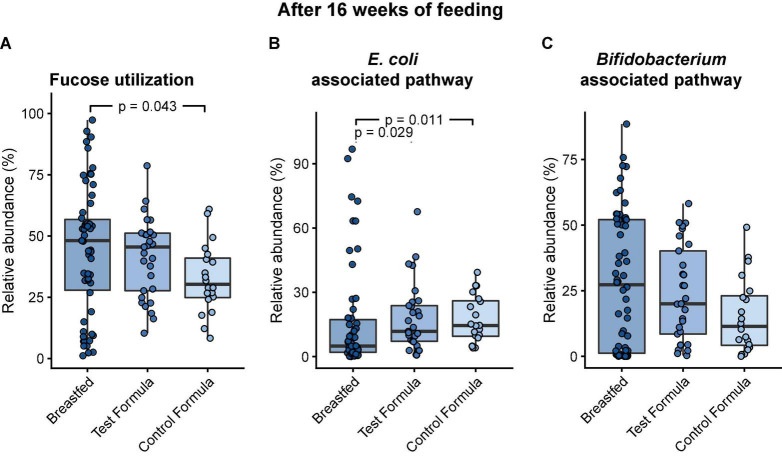
Box plots of the relative abundance of MGS that carry the functional potential to grow on fucose after 16 weeks of feeding in infants in the three feeding groups: Breastfed, test formula, and control formula. Comparisons between feeding groups were carried out for the total abundance of MGS with the functional potential to grow on fucose **(A)**, MGS carrying the pathway annotated in *E. coli* **(B)**, and MGS carrying the pathway annotated in *B. breve* **(C)**. Horizontal lines indicate the median; box boundaries indicate the interquartile range; whiskers represent values within 1.5 × the interquartile range of the first and third quartiles. Statistics: Mann–Whitney *U*-test. *P*-values < 0.05 are shown.

Fucose catabolism was evaluated in pathways first described in *Bifidobacterium breve* (20, 27) and was contrasted to pathways common to Clostridia and *E. coli* (19). There was no significant difference between the formula groups in the functional potential of the infant gut microbiome to grow on fucose, but there was a significant difference between breast-fed and control formula groups (Figure 5A). However, formula feeding was associated with a significant increase in the *E. coli*-associated pathway (test formula, *p* = 0.029; control formula, *p* = 0.011; Figure 5B) and a decrease, although not significant, in the *Bifidobacterium*-associated pathway (Figure 5C).

**FIGURE 6:**
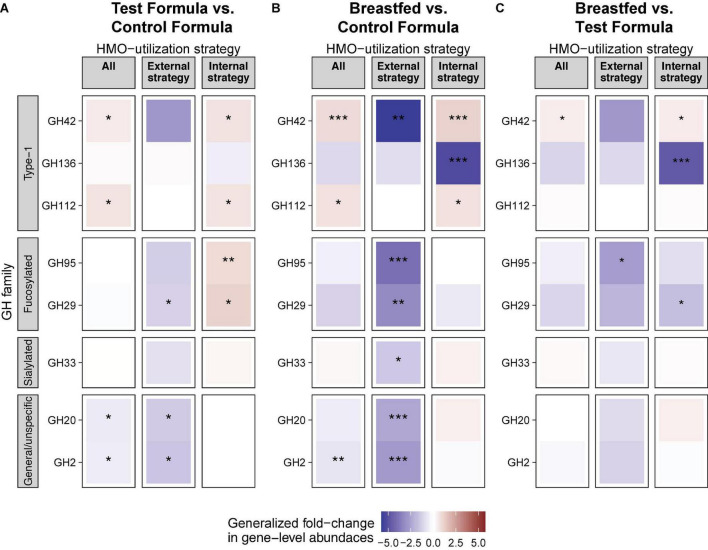
HMO is associated with differential abundance of HMO utilization genes in the gut. Generalized fold change in gut microbiota gene-level abundances of glucosyl hydrolase (GH) between feeding groups at week 15. Genes were assigned as “external strategy” if they encoded a transmembrane or secretory signal peptide. Genes without a signal peptide were assigned to the group “internal strategy.” “All” includes all genes. GH families were grouped based on their affinity toward specific groups of HMOs. Statistics: Mann–Whitney *U*-test. *P*-value cutoffs: *p < 0.05, **p < 0.01, and ***p < 0.001.

Degradation of HMOs in breastmilk requires the activity of several different enzymes, which are best characterized in *Bifidobacterium* (21). In *Bifidobacterium*, glycosyl hydrolases with known activity on HMOs belong to, in addition to GH95, GH42, GH136, GH112, GH29, GH33, GH20, and GH2 (21) and may be expressed externally or internally. The overview of the differences in the gene-level abundances of GH families between formula groups after 16 weeks of feeding are shown in Figure 6.

A total of two GH families (GH42 and GH112) were significantly increased, while two other GH families (GH20 and GH2) were significantly decreased in the test formula groups compared to the control formula group (*p* < 0.05, Figure 6A). All of these were also significantly different (*p* < 0.05) between the breastfed and control formula groups (Figure 6B), while only one of these, GH42, was significantly different (*p* < 0.05) between the breastfed and test formula groups (Figure 6C). In addition, GH genes annotated to the “internal” HMO strategy were generally increased in both the breastfed and test formula groups compared to the control formula group. Conversely, GH genes annotated to the “external” HMO strategy were overall decreased in the breastfed and test formula groups compared to the control formula group. Internal GH29, which cleaves fucose from α-*1,3*-fucosulated HMOs, was also increased in the test formula compared to the control formula group, but not in the breastfed group. The external fucosidases annotated to GH29 were increased in the control formula compared to the test formula and breastfed groups (*p* < 0.05). Gene expression for type-1 HMO linkages gave the strongest differentiation between breastfed and formula-fed infants and were more apparent for metabolism within the microbes than for enzymes associated with extracellular activity. The relative abundance of GH33, directed against sialylated carbohydrates, was not much different across feeding groups. Non-specific hydrolyses GH20 and GH2 were less expressed in the BF group than in either formula groups in the external strategy, and the 2′FL group was reduced compared to the control in the external strategy as well.

We did not find any differences in microbiome gene expression as a function of maternal or infant secretor status.

## Discussion

There were no notable findings in the growth data; average daily weight gain did not differ between formula groups. This absence of difference between formula groups and the slightly greater weight gain of formula-fed infants compared to that of breastfed infants are essentially mean differences calculated in a large review of similar studies (25). ITT results were consistent with per protocol results, evidence that attrition did not affect the study outcome. Similarly, there were no differences in the length of head circumference gains of the two formula groups.

We found no effect of enrollment age on weight gain of formula-fed subjects, although the range of ages was modest, that is, < 15 days, or 2–3 weeks or 3–4 weeks. There appeared to be an emerging difference in weight gain between formula-fed infants and breastfed infants when infants were enrolled after 3 weeks of age, although not in the overall sample, which is consistent with more rapid weight gain of breastfed infants than formula-fed infants in the first few weeks of life. Our data on the small modification of carbohydrate composition support enrollment at ages older than 2 weeks, which is specified by regulation 21 CFR 106.96(b)(1), although it is possible other nutrient modifications could affect growth selectively in either the first 2 weeks or between 2 and 4 weeks of life.

Safety data indicated no excess of AEs reported for the 2′FL group, which is unsurprising given previous studies on 2′FL (3, 4, 28). The greater number if AEs reported in the U.S. infants may be a cultural factor. Despite comparable training, U.S. investigators may be more likely to classify any symptom as an AE than Honduras investigators.

The microbiome results indicated 2′FL addition had a little effect on the overall taxonomic composition or diversity of the microbiome, at the same time confirming previous evidence of substantial differences between breast-fed and formula-fed infants after feeding for 16 weeks (29). Among all infants, vaginal delivery resulted in higher diversity and richness than cesarean delivery at the initial sampling, but the differences by birth manner were not significant in the diet groups for week 16 sample, indicating that our methods were sufficiently sensitive to detect physiologic effects of the birth mode (30) and subsequent feeding (29). Other studies also have reported that differences in the microbiome composition between infants born vaginally or by cesarean section are most profound within the first month of life (31, 32). The few differences in the functional potential of GMMs between the formula groups were not really surprising, given the modest taxonomical differences. Our 2′FL level was based on a mean value for human milk but might have been too low to generate many differences. The 2′FL may not produce effects at the level of richness or diversity; others have reported among infants exclusively breastfed, there is no effect on overall microbiome as a function of maternal FUT2 status (33).

We were able to show effects of 2′FL addition on the relative abundance of genes involved in carbohydrate metabolism, which has been presumed (34) but not previously demonstrated to occur *in vivo*. Importantly, we were able to show an increase in the gene-level abundance of α-1,2-fucosidases (GH95 family) without a signal peptide in the test formula compared to the control formula group, indicating that the addition of 2′FL to the formula may increase the functional potential of the microbiome to degrade α-1,2-fucoslyalted HMOs internally.

The significant difference between test and control groups in GH42 for the external strategy may reflect greater utilization of type 1 carbohydrates such as derived from mucin, not specifically 2′FL, such as derived from mucin. Also, the “external strategy” occurs in Bacteroidetes, which were elevated in the control group (35). The modest increase in the relative abundance of GH95 in controls compared to test or breastfed infants was observed only for the external strategy (Figure 6), which is paradoxical, as one would expect increased substrate to produce more abundant catabolic capacity. Perhaps that occurred by chance or was secondary to increased Bacteroidetes. We did not notice increased relative abundance of Clostridia (Supplementary Table 2), although others have reported this after feeding 2′FL (10). Overall, for each of the subsets of glycosyl hydrolyases (type 1, fucosylated, or sialylated carbohydrates, or for non-specific hydrolyses), the differences between breastfed and test formula infants were less pronounced than the differences between breastfed and control infants. This occurred with GOS and FOS in both formulas, supporting a specific effect of 2′FL. While the addition of 2′FL generated a pattern closer to that of breastfed infants, substantial gaps between breast-fed and formula-fed infants remain.

We had hoped to correlate 2′FL with reports of AEs including the incidence of infectious disease, but the number of AEs and number of microbiome samples were insufficient for that analysis. Barton et al. reported significant associations of *FUT2* SNPs rs601338 and rs602662 with the risks of reported respiratory and diarrheal illnesses, as well as vomiting and nocturnal cough, from birth to 24 months of age in infants in a population study (36). However, neither the maternal secretor status (FUT2 gene) did not independently correlate to reduced risk of infant respiratory or GI infection nor the maternal *FUT2* genotype found to be a significant predictor of infant health outcomes when the infant *FUT2* genotype was included in the model. Given the evidence of enhanced gene abundance in response to dietary 2′FL, further experiments could be powered to detect 2′FL protection against infection and provide direct evidence of augmented catabolism of luminal 2′FL, particularly among infants negative for FUT2.

A limitation with the shotgun metagenomic sequencing is that the relative abundance is determined, and there could be a difference in the bacterial load among feeding groups. Although we did not detect a significant difference in the relative abundance of *Bifidobacterium* among formula groups, breastfed infants generally had a higher relative abundance of *Bifidobacterium* (Supplementary Table 2), which is in line with previous studies (37). The differences in the abundance of *Bifidobacterium* between formula-fed and breastfed infants may explain some differences in gene abundances between these groups. Bacteroidetes increase in response to the complementary diet (38), which is delayed in breastfed infants. Our data showing increased capacity to degrade 2′FL do not demonstrate that such degradation does in fact occur. Measuring the levels of 2′FL in the stool would allow investigating if the functional potential of the infant gut microbiome to utilize 2′FL correlates negatively with the concentration of 2′FL in stool.

In conclusion, the addition of a physiologic level of 2′FL had no effect on growth or incidence of adverse effects of formula-fed infants, adding evidence of safe use of this HMO in the infant formula. The addition of 2′FL resulted in modest changes in microbiome in the direction of breastfed infants, consistent with more metabolic capability of HMOs by *Bifidobacterium.*

## Data availability statement

The sequencing data presented in the study are deposited in the European Nucleotide Archive (ENA), accession number PRJEB54991. Sample overview and metadata is found in Supplementary Table 4.

## Ethics statement

The studies involving human participants were reviewed and approved by the WIRB-Copernicus Group Central IRB 202 Carnegie Center Suite 107 Princeton, NJ 08540. Written informed consent to participate in this study was provided by the participants’ legal guardian/next of kin.

## Author contributions

JW, PN, and CB: conceptualization. JW and PN: methodology, analysis, and writing. JW: original draft preparation. All authors have read and agreed to the published version of the manuscript and contributed to the article and approved the submitted version.

## Footnotes

1 We have capitalized Breastfed when referring to the study group; and lower case when referring to infant feeding in general.
